# Supplementary material for: Quantum phase transitions in highly crystalline two-dimensional superconductors
Source: Nat Commun. 2018 Feb 22;9:778. doi: 10.1038/s41467-018-03275-z (PMC5823914; doi:10.1038/s41467-018-03275-z)
Supplement: Supplementary file 1 — Supplementary Information [file 41467_2018_3275_MOESM1_ESM.pdf]

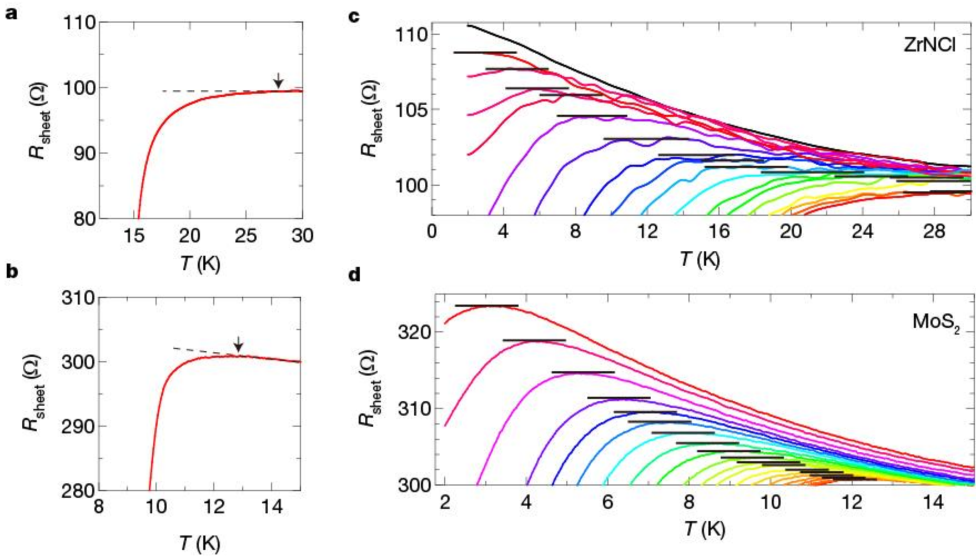

Supplementary Figure 1 | Close-ups of the sheet resistance as a function of temperature for ion-gated ZrNCl and MoS_2. a, b, Sheet resistance as a function of temperature at zero magnetic field for ZrNCl (**a**) and MoS_2 (**b**). The black dashed line is a linear fitting before the superconducting transition. The arrows show T_{onset} at zero magnetic field. **c**, **d**, Sheet resistance as a function of temperature in out-of-plane magnetic fields for ZrNCl (**c**) and MoS_2 (**d**). The applied magnetic fields are 0.1, 0.2, 0.3, 0,5 and 0.7 T, and vary in 0.3 T steps from 0.9 T to 1.8 T and in 0.5 T steps from 2 T to 5 T, and 7 and 8.5 T for ion-gated ZrNCl. They are 0.05, 0.1, 0.2 and 0.4 T, and vary in 0.3 T steps from 0.6 T to 3 T, in 0.5 T steps from 3.5 T to 6 T and in 1 T steps from 7 T to 9 T for ion-gated MoS_2.

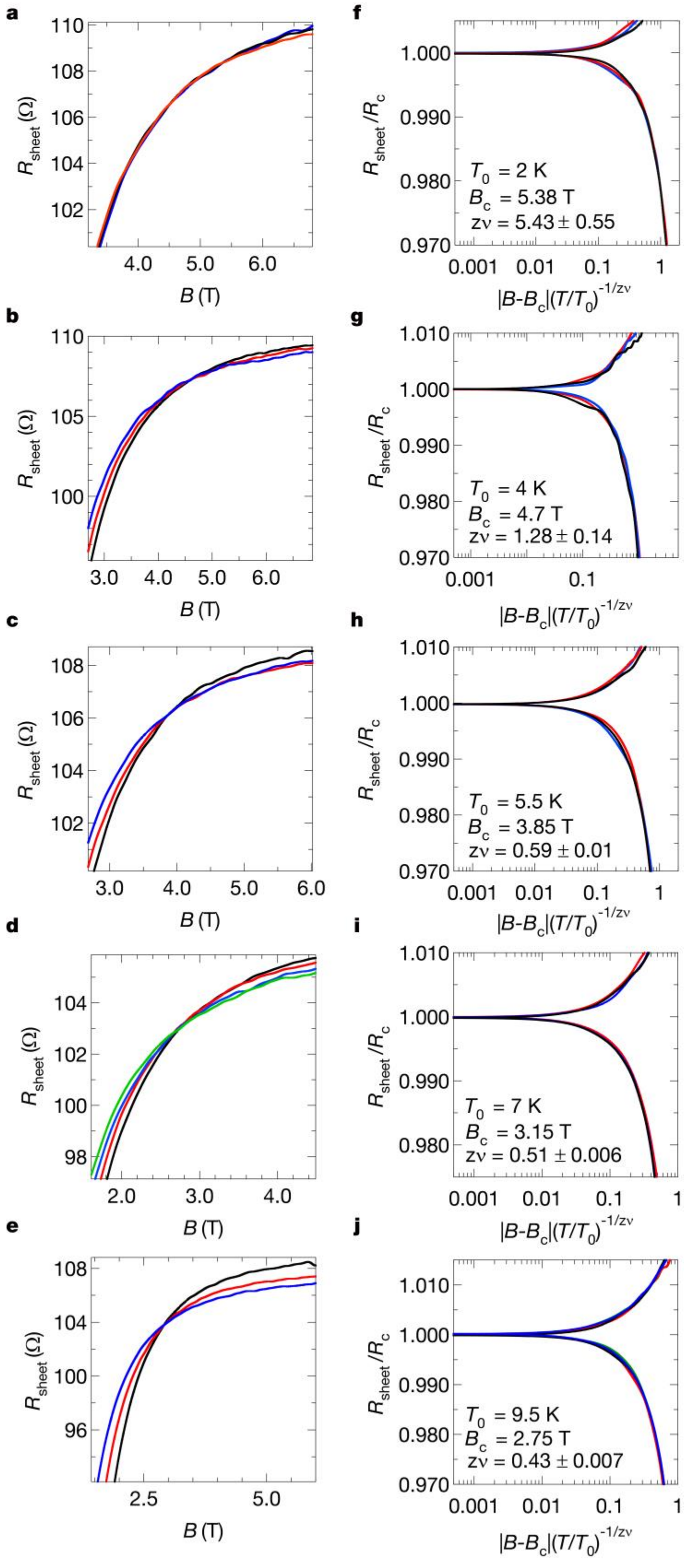

Supplementary Figure 2 | FSS analysis at different temperatures for ion-gated ZrNCl. a–**e**, Sheet resistance as a function of magnetic field at different temperature intervals. **f**–**j**, Normalized R_{sheet} as a function of the scaling variable $|B\text{-}B_c|(T/T_0)^{-1/z\nu}$. Here, the B_c determined the crossing points of $R_{sheet}(B)$ curves, and T_0 is the lowest temperature in each set of the R-B curves.

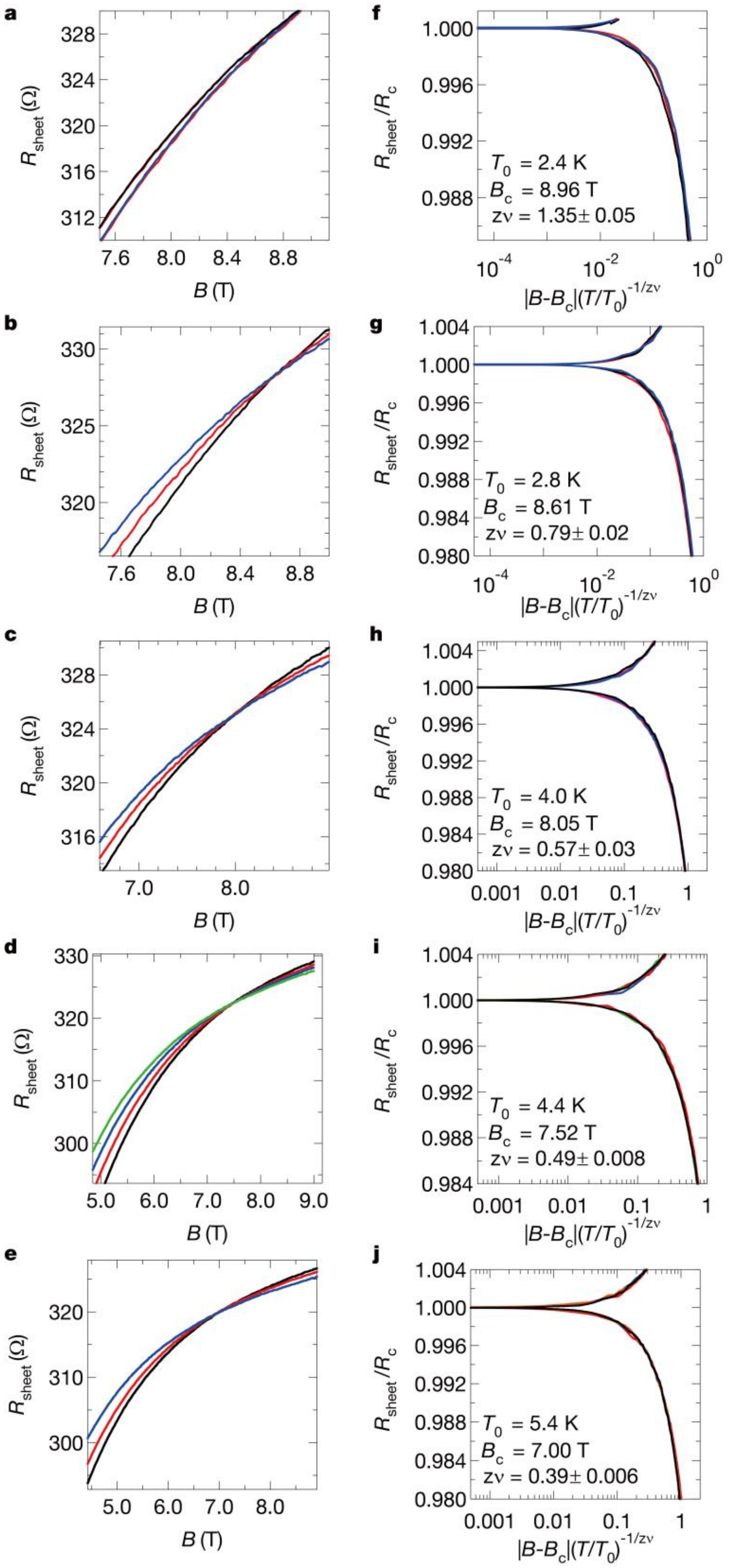

Supplementary Figure 3 | FSS analysis at different temperatures for ion-gated MoS_2. a–e, Sheet resistance as a function of magnetic field close to the transition boundary at different temperatures. **f–j**, Normalized R_{sheet} as a function of the scaling variable $|B-B_c|(T/T_0)^{-1/z\nu}$. Here, the B_c determined the crossing points of $R_{sheet}(B)$ curves, and T_0 is the lowest temperature in each set of the *R-B* curves.

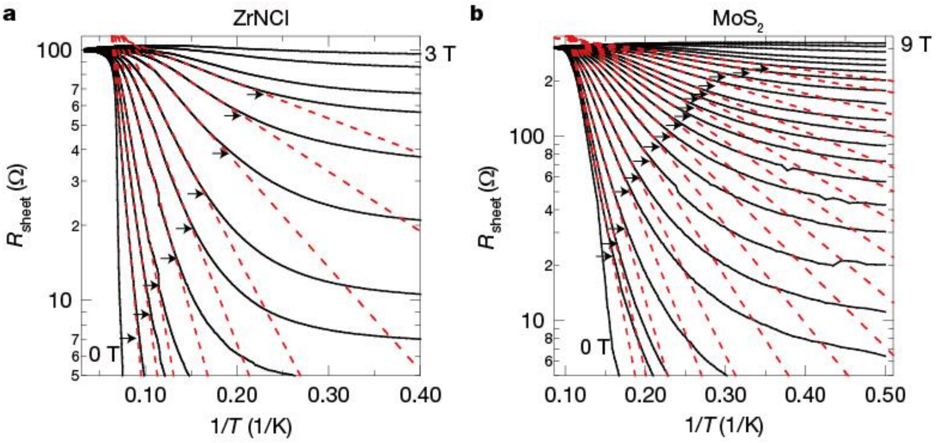

Supplementary Figure 4 | Arrhenius plot of the sheet resistance of ion-gated ZrNCl and MoS_2. **a**, For ion-gated ZrNCl, the applied magnetic fields vary in 0.1 T steps from 0 to 0.3 T, in 0.2 T steps from 0.5 to 0.9 T, in 0.3 T steps from 1.2 to 1.8 T and in 0.5 T steps from 2.0 to 3.0 T. **b**, For ion-gated MoS_2, they vary in 0.1 T steps from 0 to 0.2 T, in 0.2 T steps from 0.4 to 0.6 T, in 0.3 T steps from 0.9 to 3.0 T in 0.5 T steps from 4 to 7 T and in 1 T steps from 8 to 9 T. The red dashed lines show the activated behavior based on the 2D collective creep model described by $R_{\mathrm{sheet}} = R' \exp(-U(H)/k_{\mathrm{B}}T)$. The crossover temperature T_{cross} is defined as the temperature at the deviation point from the red dashed lines, shown by arrows.

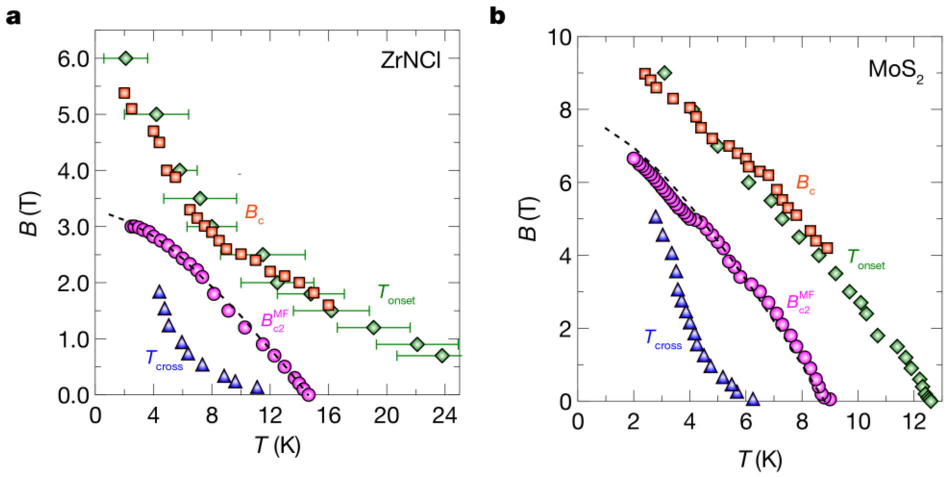

Supplementary Figure 5 | *B*-*T* phase diagram of 2D superconducting ZrNCl and MoS_2. The magnetic field –temperature phase diagram of the ZrNCl- (**a**) and MoS_2-EDLT (**b**). Orange squares show the crossing points B_c of *R*-*B* curves in Figs. 2b and d at neighboring temperatures. Pink circles show the mean field upper critical field B_{c2}^{MF} derived from the UD scaling[1]. Green diamonds show the onset of superconducting fluctuation T_{onset}. Error bars of green diamonds represent the ambiguity of T_{onset} defined by $dR_{sheet}/dT = 0$ due to the experimental resolution. Blue triangles show the crossover temperature T_{cross} from the thermal creep regime to the quantum creep regime defined as the deviation points from the linear dependence of R_{sheet} in the Arrhenius plot. Dashed curve shows the fitting of B_{c2}^{MF} (T) by the WHH theory.

Supplementary Note 1. Determination of mean-field upper critical field

A reliable determination of the mean field upper critical field $B_{c2}^{MF}(T)$ or transition temperature $T_c^{MF}(B)$ is indispensable to a proper understanding of the vortex state and various fluctuation effects, leading to the comprehensive description of the field – temperature (B-T) phase diagram. In two dimensional (2D) or highly anisotropic superconductors, however, the significant thermal fluctuation, which smears out the critical behavior at $B_{c2}^{MF}(T)$ or $T_c^{MF}(B)$ in the mean field theory, makes it difficult to obtain the appropriate value of it directly from the transport properties. For instance, the T dependence of electrical conductivity in B observed in this case is in rather continuous function of T from the normal to the vortex state without the divergence at $T_c^{MF}(B)$. It is well known that the Aslamazov and Larkin type fluctuation theory assuming only Gaussian fluctuations[2], implying no interaction between the fluctuations, fails to fit the conductivity just around $T_c^{MF}(B)$ due to this divergence.

In this work, in order to estimate $T_c^{MF}(B)$ from the temperature dependence of sheet resistance $R_{sheet}(T)$ in B, we adopted the Ullah and Dorsey theory based on the time-dependent Ginzburg-Landau equation with the Hartree approximation, which takes into account the interaction between fluctuations[3]. This interpolates smoothly between the high-temperature Gaussian fluctuation regime and the low-temperature vortex-flow regime, resulting in successful fitting the transport properties of cuprates and low-T_c films[4,5]. According to their scaling formula, the 2D conductance $G_{\rm fl}^{\rm 2D}$ due to thermal fluctuations are given using the reduced temperature $t = T/T_c^{MF}(0)$ and field $b = B/B_{c2}^{MF}(0)$ as follows[3,4],

$$G_{\rm fl}^{\rm 2D} = C_0 A_0 \left(\frac{t}{b}\right)^{1/2} \mathcal{F}\left(A_0 \frac{\epsilon_{\rm B}}{(bt)^{1/2}}\right), \qquad (1)$$

where $C_0 = \Gamma_0^{-1} m^* \xi(0)^2 / 4\pi\hbar^2 \Lambda_{T_c}$, $A_0 = \left[d_{\rm eff}\Lambda_{T_c}/(2\kappa^2 - 1)\xi^2(0)\right]^{1/2}$ and $\epsilon_{\rm B} = t - 1 + b$ with Γ_0^{-1} the scattering factor, m^* the effective mass of electron, $\xi(0)$ the coherence length at zero temperature, $\Lambda_{T_c} = \phi_0^2/4\pi\mu_0 k_{\rm B} T_{c0}$ the thermal length at T_{c0} (= $T_c^{MF}(0)$) and $d_{\rm eff}$ the effective thickness. The universal function $\mathcal{F}(x)$, which is implicitly given by

$$x = \frac{1}{\mathcal{F}(x)} + \mathcal{F}(x), \qquad (2)$$

leads to $\mathcal{F}(x) \sim x^{-1}$ for $x >> 0$ and $\mathcal{F}(x) \sim -x$ for $x << 0$. By using the GL expression of $B = B_{c2}^{\rm MF}(0)(1 - T/T_c^{\rm MF}(B))$ near $T_c^{\rm MF}(0)$, Supplementary Eq. (1) is transformed to

$$G_{\rm fl}^{\rm 2D} \left(\frac{B}{T}\right)^{1/2} = \tilde{\mathcal{F}}\left(\frac{T - T_c^{\rm MF}(B)}{(BT)^{1/2}}\right), \qquad (3)$$

where $\tilde{\mathcal{F}}(x) = C_0 A_1 \mathcal{F}(A_1 x)$ with $A_1 = A_0 (B_{c2}^{\mathrm{MF}}(0)/T_c^{\mathrm{MF}}(0))^{1/2}$. By using this scaling law for the fluctuation conductance, which is equal to $1/R_{\mathrm{sheet}}(T)$-$1/R_{\mathrm{N}}$ with R_{N} the normal sheet resistance, we can derive $T_c^{\mathrm{MF}}(B)$ as a fitting parameter. As noted in Supplementary Ref. 2 and 3, the scaling law in Supplementary Eq. (3) does not give the correct expression in the flux flow regime due to the effect of pinning and the interaction between the vortices, which are not considered in the theory. Therefore, we used the scaling at high temperature above $T_c^{\mathrm{MF}}(B)$.

Supplementary Note 2. Definition of onset of superconducting fluctuations

The resistance drop starts to occur at much higher temperature than T_c^{MF}, reflecting significant contribution of 2D superconducting fluctuation. To complete the *B*-*T* phase diagram, we determine the onset temperature of superconducting fluctuation T_{onset} from the *R*-*T* curve at zero and finite magnetic fields as following. In zero magnetic field, the onset was defined as the temperature at which the resistance deviates the linear fit (black dashed line) in the normal state. In finite magnetic fields, since the *R*-*T* data show the localized behavior, T_{onset} was defined as the temperature at which the sheet resistance becomes flat as a function of temperature, namely $dR/dT = 0$, which is indicated as black solid line in Supplementary Figure 1. T_{onset} are plotted by green diamonds in Figs. 1d and e for ZrNCl and MoS_2, respectively. Importantly, T_{onset} and B_c (plotted as orange squares) agree well with each other. This is consistent with the idea that the boundary between the superconducting and insulating behavior corresponds to the crossing point of magnetoresistance in the localized system. Thus, the plots of B_c also describe the onset of superconducting fluctuations well.

Supplementary Note 3. Application of Griffiths theory to superconductors

The diverging behavior of dynamical critical exponent is nowadays discussed within the context of the theoretical model based on the Griffiths singularity, where the rare ordered regions play a significant role for describing the quantum phase transition. The concept of the rare region was first pointed out by Griffiths in showing the nonanalytic behavior of magnetization in randomly diluted Ising ferrimagnets. In such a system, due to the random quenched disorder such as impurity and defects, the rare spatial regions that are locally in the magnetic persists in non-magnetic bulk. Although there are many theoretical works describing the Griffiths state and Griffiths singularity using lattice (Ising) systems[6–10], the related experimental works have been limited within 3*d*-ferromagnets, $Sr_{1-x}Ca_xRuO_3$[11], $Ni_{1-x}V_x$.[12] and 4*f* electron systems

The possibility of the Griffiths singularity in superconducting systems has been discussed in ultrathin disordered nanowires[13], but there are no example in 2D systems. Very recently, the quantum Griffiths singularity is experimentally observed in two-kinds of 2D superconductors: Ga thin films[14] and $LaAlO_3/SrTiO_3$ (110) polarized-interface[15]. In these two systems, multiple critical points were observed as is reported in our study. To analyze the experimental data, they used the finite size scaling analysis for different temperature regimes (which is explained in Supplementary Note 4), and they found the dynamical critical exponent shows a power-law form. This anomalous scaling behavior, as is observed in the present study, is suggested in the quantum random transverse field Ising model[8,9], which shows the activated scaling with continuously varying dynamical critical exponent *z*, when approaching the infinite-randomness QCP. Such critical behavior originates from the special condition of disorder in the statistical treatment, where the average strength of disorder (magnitude of inhomogeneity) increases without limit under coarse graining, that is theoretically equivalent to $d\nu < 2$ (violation of the Harris criterion[7]) with *d* the system dimension and ν the correlation length exponent. Therefore, the Griffith state with the diverging *z* should satisfies this condition. Indeed, ν is theoretically predicted as 0.5 for the superconductor-metal transition in a clean 2D superconducting system[16], the Harris criterion is violated with $d\nu = 1$. In this case, the introduction of finite quenched disorder can lead to the infinite-randomness QCP, observed as the quantum Griffith singularity. Following this scenario, the product of the critical exponents $z\nu$ is expressed by the activated scaling law[13,17–19]:

$$z\nu \approx C(B - B_c^*)^{-\upsilon\Psi} \tag{4}$$

with the constant *C* and the 2D infinite-randomness critical exponents of $\upsilon \approx 1.2$ and $\Psi \approx 0.5$[20,21]. Here, B_c^* is infinite randomness critical point. This formula is in good agreement with the experimental data of Ga, LAO/STO and our systems.

Supplementary Note 4. Finite-size scaling analysis around QPT at different temperature regimes

To investigate the multiple-crossing behavior of magnetoresistance shown in Fig. 2 in the main text, and to test the possible application of quantum Griffiths singularity, we performed the finite size scaling (FSS) law of magnetoresistance for QPT expressed as,

$$R(B,T) = R_{\mathrm{c}} f\left(\frac{B - B_{\mathrm{c}}}{(T/T_0)^{1/z\nu}}\right) \tag{5}$$

in various temperature regimes around the crossing points based on the data shown in Figs. 2a and d (main text). Here, R_c and B_c, are the asymptotic critical sheet resistance and, the critical magnetic field defined as the values at the crossing points, T_0 the lowest temperature used in the scaling of R-B curves, $f(x)$ the scaling function with $f(0) = 1$, z and ν the dynamic and static critical exponent, respectively. To investigate the evolution of the effective critical exponents $z\nu$ with the decrease (or increase) in T (or B), five representative crossing points were selected both for ZrNCl and MoS_2, and then the FSS analysis in the narrow temperature regimes around these crossing points were performed. During the scaling, we derived $z\nu$ by assuming that the functional forms of $f(x)$ both for $x > 0$ and $x < 0$ unchanged. In Supplementary Figures 2 and 3, the left graphs show the sheet resistance as a function of magnetic field close to the selected crossing points. The right graphs shows normalized R_{sheet} as a function of the scaling variable $|B$-$B_c|(T/T_0)^{-1/z\nu}$.

The obtained values of $z\nu$ are plotted as a function of B as shown in Figs. 3a and b in the main text, respectively. $z\nu$ is not constant and varies as a function of magnetic field (temperature) in both systems, and seems to diverge toward a certain critical field following the relation of $z\nu \sim (B - B_c^*)^{-0.6}$, which is consistent with Supplementary Eq. (4). These results can be the evidence for occurrence of quantum Griffiths singularity at $B = B_c^*$.

Supplementary Note 5. Quantum metallic states and their crossover to thermal vortex creep

In our previous paper[22], we found that the zero-resistance state in gate-induced 2D superconductivity in ZrNCl is easily destroyed, once the out-of-plane magnetic field is switched on. In the low temperature region, the resistance becomes finite and independent of temperature. Such a state is regarded as a quantum metallic state[22], possibly ascribed to the extremely weak pinning and large quantum fluctuations due to the 2D structure. The characteristic feature of 2D superconductors with weak pinning is that the zero-resistance Meissner state is not discernible and a wide region of *B*-*T* phase diagram is covered by this quantum metallic state, where the energy dissipation occurs though the quantum creep of vortices, and thus this state may be regarded as vortex liquid[22].

The quantum metallic state (quantum creep of vortices) is clearly distinguished from the thermal creep state, through the following analysis. Supplementary Figure 4 displays the Arrhenius plot of temperature dependence of sheet resistance. The high temperature regions are well fitted by the thermal activation-type behavior both for ion-gated ZrNCl and MoS_2 (red dashed lines):

$$R(T) = R'\exp\left(-U(H)\middle/k_{\mathrm{B}}T\right). \qquad (6)$$

Here $U(H)$ is the activation energy and R' is the fitting parameter. This indicates that the vortex motion is governed by the thermally-activated 2D collective creep[23].

On the other hand, in the low temperature region, the resistance deviates from the activation behavior and tends to saturate, displaying a crossover from thermal to quantum creep regions. The crossover temperature T_{cross} is defined as the temperature, where the experimental data deviates from the red dashed line (shown by arrows in Supplementary Figure 4).

Based on the transport data including the analysis with the 2D collective creep model[23], the UD scaling model[1], and of the multiple-crossing points, we provide the *B*-*T* phase diagram of 2D superconducting ZrNCl and MoS_2 in Supplementary Figure 5. Dashed curve shows the fitting by Wertharmer-Helfand-Hohenberg (WHH) theory[24]. Orange squares show the crossing points B_{c} of *R*-*B* curves in Figs. 2b and d at neighboring temperatures. Pink circles show the mean field upper critical field $B_{\mathrm{c2}}^{\mathrm{MF}}$ derived from the UD scaling[1]. Green diamonds show the superconducting onset T_{onset} Blue triangles show the crossover temperature T_{cross} from the thermal creep regime to the quantum creep regime as the deviation points from the linear dependence of *R* in the Arrhenius plot.

Supplementary Note 6. Crossover from quantum metallic state to quantum Griffiths state

Figure 4c in the main text is suggesting that the quantum fluctuation governs the phase evolution particularly at low temperature. The crossover from the quantum metallic state to the Griffiths state is rationalized as follows. Although the ground state predicted in disorder-free 2D superconductors is the vortex lattice phase[25], which is similar to the 3D disorder-free type-II superconductors[26,27], the long-range order of a vortex lattice easily becomes unstable because of the fluctuations caused by disorder in the absence of the longitudinal elasticity of vortices. This leads to the imperfect vortex lattice state containing many dislocations even in relatively clean system. At low magnetic fields, the plastic motion of dislocations based on the weak pinning and weak elasticity occurs by the thermal[23] and quantum fluctuation in high and sufficiently low temperatures, respectively, as shown in Figure 4c in the main text. In this situation, the sample consists of the superconducting puddles with short-range ordered vortices and the dissipation regime surrounding them at low temperatures. With increasing magnetic field above B_{c2}^{MF}, the dissipation region evolves into the normal state, but it is possible that puddle-like superconducting islands remain at very low temperature because of the effect of the quantum fluctuation stabilized by quenched disorder, resulting in the quantum Griffiths state with rare superconducting regions (Fig. 4c). Thus, the quantum creep region (quantum metallic state) is naturally connected to the quantum Griffiths state at low temperature. This situation can be a consequence of strong quantum fluctuation in the 2D superconductor with the very weak but finite pinning effect, leading to a standout effect of randomness.

The difference of quantum Griffiths state and quantum metallic state is considered to originate from two kinds of fluctuation: the amplitude and phase fluctuation, using the analogy to the case of the thermal fluctuations. In other words, the quantum Griffiths state and quantum metallic state correspond to quantum fluctuations of amplitude and phase of order parameter, respectively. In the quantum metallic state, the individual motion of vortices is suppressed by the elasticity of the vortices (in other words, the vortices locally form lattices), and vortex creep occurs at the connecting region of local lattices, where the elastic energy of vortices is small. Therefore, the phase fluctuation locally occurs in the quantum metallic state. With increasing magnetic field, the vortex-lattice mismatch regions, where the quantum creep frequently occurs, widen and eventually become normal states. However, there remain the locally ordered regions due to the pinning by quenched disorder, which form the puddle-like regions with nonzero order parameter. This situation may evolve into to a quantum Griffiths state with spatial amplitude fluctuation of order parameter.

Supplementary References

1. Ullah, S. & Dorsey, A. T. Critical fluctuations in high-temperature superconductors and the Ettingshausen effect. *Phys. Rev. Lett.* **65,** 2066–2069 (1990).
2. Aslamasov, L. G. & Larkin, A. I. The influence of fluctuation pairing of electrons on the conductivity of normal metal. *Phys. Lett. A* **26,** 238–239 (1968).
3. Ullah, S. & Dorsey, A. T. Effect of Fluctuations on the transport properties of type-II superconductors in a magnetic field. *Phys. Rev. B* **44,** 262–273 (1991).
4. Theunissen, M. H. & Kes, P. H. Resistive transitions of thin film superconductors in a magnetic field. *Phys. Rev. B* **55,** 15183–15190 (1997).
5. Palstra, T., Batlogg, B., Schneemeyer, L. & Waszczak, J. Transport entropy of vortex motion in $YBa_2Cu_3O_7$. *Phys. Rev. Lett.* **64,** 3090–3093 (1990).
6. Vojta, T. & Hoyos, J. A. Criticality and quenched disorder: Harris criterion versus rare regions. *Phys. Rev. Lett.* **112,** 75702 (2014).
7. Harris, A. B. Effect of random defects on the critical behaviour of Ising models. *J. Phys. C Solid State Phys.* **7,** 1671 (1974).
8. Fisher, D. S. Random transverse field Ising spin chains. *Phys. Rev. Lett.* **69,** 534–537 (1992).
9. Fisher, D. S. Critical behavior of random transverse-field Ising spin chains. *Phys. Rev. B* **53,** 1689–1699 (1995).
10. Motrunich, O., Mau, S.-C., Huse, D. & Fisher, D. Infinite-randomness quantum Ising critical fixed points. *Phys. Rev. B* **61,** 1160–1172 (2000).
11. Demkó, L. *et al.* Disorder promotes ferromagnetism: rounding of the quantum phase transition in $Sr_{1-x}Ca_xRuO_3$. *Phys. Rev. Lett.* **108,** 185701 (2012).
12. Ubaid-Kassis, S., Vojta, T. & Schroeder, A. Quantum Griffiths phase in the weak itinerant ferromagnetic alloy $N_{1-x}V_x$. *Phys. Rev. Lett.* **104,** 066402 (2010).
13. Del Maestro, A., Rosenow, B., Hoyos, J. A. & Vojta, T. Dynamical conductivity at the dirty superconductor-metal quantum phase transition. *Phys. Rev. Lett.* **105,** 145702 (2010).
14. Xing, Y. *et al.* Quantum Griffiths singularity of superconductor-metal transition in Ga thin films. *Science* **350,** 542–545 (2015).
15. Shen, S. *et al.* Observation of quantum Griffiths singularity and ferromagnetism at the superconducting $LaAlO_3/SrTiO_3$ interface. *Phys. Rev. B* **94,** 144517 (2016).
16. Sachdev, S., Werner, P. & Troyer, M. Universal conductance of nanowires near the superconductor-metal quantum transition. *Phys. Rev. Lett.* **92,** 237003 (2004).

17. Hoyos, J., Kotabage, C. & Vojta, T. Effects of dissipation on a quantum critical point with disorder. *Phys. Rev. Lett.* **99,** 230601 (2007).
18. Vojta, T., Kotabage, C. & Hoyos, J. Infinite-randomness quantum critical points induced by dissipation. *Phys. Rev. B* **79,** 024401 (2009).
19. Del Maestro, A., Rosenow, B., Müller, M. & Sachdev, S. Infinite randomness fixed point of the superconductor-metal quantum phase transition. *Phys. Rev. Lett.* **101,** 035701 (2008).
20. Kovács, I. & Iglói, F. Renormalization group study of the two-dimensional random transverse-field Ising model. *Phys. Rev. B* **82,** 054437 (2010).
21. Vojta, T., Farquhar, A. & Mast, J. Infinite-randomness critical point in the two-dimensional disordered contact process. *Phys. Rev. E* **79,** 011111 (2009).
22. Saito, Y., Kasahara, Y., Ye, J., Iwasa, Y. & Nojima, T. Metallic ground state in an ion-gated two-dimensional superconductor. *Science* **350,** 409–413 (2015).
23. Feigel'man, M. V., Geshkenbein, V. B. & Larkin, A. I. Pinning and creep in layered superconductors. *Phys. C Supercond. its Appl.* **167,** 177–187 (1990).
24. Werthamer, N. R., Helfand, E. & Hohenberg, P. C. Temperature and purity dependence of the superconducting critical field, H_{c2}. III. Electron spin and spin-orbit effects. *Phys. Rev.* **147,** 295–302 (1966).
25. Fisher, D. S. Flux-lattice melting in thin-film superconductors. *Phys. Rev. B* **22,** 1190–1199 (1980).
26. Fisher, D. S., Fisher, M. P. A. & Huse, D. A. Thermal fluctuations, quenched disorder, phase transitions, and transport in type-II superconductors. *Phys. Rev. B* **43,** 130–159 (1991).
27. Blatter, G., Feigel'Man, M. V., Geshkenbein, V. B., Larkin, A. I. & Vinokur, V. M. Vortices in high-temperature superconductors. *Rev. Mod. Phys.* **66,** 1125–1388 (1994).
